# Exit from Mitosis in Budding Yeast: Protein Phosphatase 1 is Required Downstream from Cdk1 Inactivation

**DOI:** 10.21203/rs.3.rs-2787001/v1

**Published:** 2023-04-12

**Authors:** Jason M. Keaton, Benjamin G. Workman, Linfeng Xie, James R. Paulson

**Affiliations:** Acacia Safety Consulting, Inc; University of Wisconsin–Oshkosh; University of Wisconsin–Oshkosh; University of Wisconsin–Oshkosh

**Keywords:** Cdc28, Cdk1/cyclin B, cell cycle, mitosis, mitotic exit, MPF, rebudding

## Abstract

We show that inactivation of the protein kinase Cdk1/Cyclin B (Cdc28/Clb 2 in the budding yeast *Saccharomyces cerevisiae*) is not only *necessary* for cells to leave mitosis, as is well known, but also *sufficient* to trigger mitotic exit. Cells carrying the mutation *cdc28*-*as1*, which makes Cdc28 (Cdk1) uniquely sensitive to the ATP analog 1NM-PP1, were arrested with spindle poisons and then treated with 1NM-PP1 to inhibit Cdk1. This treatment caused the cells to exit mitosis and enter G1-phase as shown by initiation of rebudding (without cytokinesis), production of “shmoos” (when α-factor was present), stabilization of Sic1, and degradation of Clb2. This result provides a system in which to test whether particular gene products are required downstream from Cdk1 inactivation in exit from mitosis. In this system, the mutation *cdc28*-*as1* is combined with a conditional mutation in the gene of interest. Using this approach, we demonstrate that Protein Phosphatase 1 (PPase1; Glc7 in *S. cerevisiae*) is required for reestablishment of G1-phase following Cdk1 inactivation. This system could be used to test whether other protein phosphatases are also needed downstream from Cdk1 inactivation, and it could be combined with phosphoproteomics to gain information about the substrates those phosphatases act on during mitotic exit.

## Introduction

The onset of mitosis in all eukaryotes is triggered in late G2-phase of the cell cycle by activation of the protein kinase Cdk1/Cyclin B, also known as M-phase Promoting Factor (MPF) (Norbury and Nurse 1992). This is a heterodimeric complex of Cyclin-dependent Kinase 1 (Cdk1; called Cdc28 in the budding yeast *Saccharomyces cerevisiae*) and a regulatory subunit, Cyclin B (Clb2 in *S. cerevisiae*). Cdk1/Cyclin B activation leads, directly or indirectly, to the phosphorylation of many proteins (e.g., Gurley et al. 1978; Stukenberg et al. 1997; Ptacek et al. 2005; Mochida and Hunt 2012; Ohta et al. 2016) and evidence suggests that these phosphorylations induce the dramatic cellular changes that characterize mitosis. These include nuclear envelope breakdown (e.g., Ward and Kirschner 1990; Heald and McKeon 1990; Peter et al. 1990, 1991), nuclear pore complex disassembly (e.g., Laurell et al. 2011), and cessation of transcription during mitosis (e.g., Gottesfeld and Forbes, 1997).

At the end of mitosis, Cdk1/Cyclin B is inactivated by destruction of its cyclin component. This occurs when the Anaphase Promoting Complex (APC), a specific ubiquitin ligase, ubiquitinates Cyclin B, marking it for destruction by the proteasome (King et al. 1995; Hixon and Gualberto, 2000). Inactivation of Cdk1 presumably leads to dephosphorylation of many mitotic phosphoproteins and reversal of the cellular changes that took place at the onset of mitosis.

Much of the published work on exit from mitosis has centered on the mechanisms by which the APC is activated, anaphase is initiated, and Cdk1/Cyclin B is inactivated. In this paper, we focus instead on the events that occur “downstream” from Cdk1 inactivation. Here “exit from mitosis” will refer to the actual processes of dephosphorylating mitotic phosphoproteins, exiting mitosis, and returning to the interphase state.

Inactivation of Cdk1 is necessary for a cell to leave mitosis. Elegant experiments show that if MPF remains active, for example if Cyclin B cannot be degraded, the cell cannot progress to G1-phase (e.g., Murray et al. 1989; Ghiara et al. 1991; Luca et al. 1991; Gallant and Nigg 1992; Luo et al. 1994; Rimmington et al. 1994; Wäsch and Cross 2002; Skoufias et al. 2007).

However, evidence suggests that inactivation of Cdk1/Cyclin B is not only *necessary* for exit from mitosis but also *sufficient* to trigger mitotic exit, provided downstream factors are functional. A variety of methods and systems have been used to inactivate or inhibit Cdk1 in metaphase-arrested cells and they invariably trigger a return to interphase (reviewed by Paulson 2007). Of course, proving the hypothesis of sufficiency with absolute certainty is difficult. One must establish that the method used to inactivate Cdk1 has not fortuitously triggered other events, independent of Cdk1 inactivation, that are also required for mitotic exit.

For a definitive test of the sufficiency hypothesis, we have used a system that allows very specific inhibition of Cdk1: *S. cerevisiae* carrying the *cdc28*-*as1* mutation (Bishop et al. 2000). This mutation makes the Cdc28 kinase (Cdk1) specifically sensitive to inhibition by a substituted pyrazolopyrimidine ATP analog (abbreviated 1NM-PP1). The use of such analog-sensitive protein kinase mutations is a general method developed in the lab of Kevan Shokat (Bishop et al. 1999, 2000; Lopez et al. 2014), which has been applied to the study of protein kinases in yeast (e.g., Dischinger et al. 2008; Cadou et al. 2010, 2013) as well as mammalian cells (e.g., Morgan et al., 2008; Haan et al. 2011; Al-Ghabkari et al. 2018). It has also been used to synchronize entry of vertebrate cells into mitosis to study the formation of mitotic chromosomes (Gibcus et al. 2018; Samejima et al. 2022).

The work reported here was motivated by two questions: Does specific inhibition of Cdc28-as1 in metaphase-arrested *Saccharomyces cerevisiae* induce the transition from mitosis to G1-phase? And if so, can this be exploited to identify genes and proteins that are required downstream from Cdk1 inactivation during exit from mitosis?

We find that treatment of metaphase-arrested cdc28-as1 cells with the ATP analog 1NM-PP1 does indeed trigger exit from mitosis, providing strong support for the hypothesis that Cdk1 inactivation is sufficient to induce exit from mitosis. This result also gives us a system with which to study events downstream from Cdk1 inactivation, independently of anaphase, chromosome segregation and cytokinesis. Using this system and a conditional mutation in Protein Phosphatase 1 (PPase 1), we show that PPase 1 (Glc7 in budding yeast; Feng et al. 1991) is required downstream from Cdk1 inactivation during exit from mitosis.

## Materials And Methods

### ATP Analog and Other Chemicals:

Benomyl (methyl 1-(butylcarbamoyl)-2-benzimidazole carbamate) was obtained from Aldrich Chemical Company (Milwaukee, Wisconsin), prepared as a 30 mg/mL stock solution in dimethyl sulfoxide (DMSO) and stored at − 20°C. α-Factor was obtained from Zyma Research (Orange, California) as a 10 mM solution in 0.1 M sodium acetate pH 5.2. Nocodazole was obtained from Sigma, prepared as a 5 mg/mL stock solution in DMSO and stored in aliquots at − 20°C. The ATP analog 4-amino-1-*tert*-butyl-3-(1′-naphthylmethyl) pyrazolo[3,4-d]pyrimidine (CAS 221244–14-0; hereafter referred to as “the analog” or “1NM-PP1”) was synthesized from 1-naphthylacetic acid in five steps, basically as described by Hanefeld et al. (1996) except that the products of steps 2 and 4 were isolated by silica gel chromatography before proceeding. The final product was also isolated by column chromatography and then recrystallized from ethanol-water, producing a white solid that gave a single spot when analyzed by thin layer chromatography. Its identity was confirmed by ^1^H NMR, COSY, and high-resolution mass spectrometry. ^1^H NMR (270 MHz, CDCl_3_) gave peaks at 1.84 (singlet, 9H), 4.75 (singlet, 2H), 4.85 (broad singlet, 2H), 7.15 (doublet, 1H), 7.38 (triplet, 1H), 7.54 (multiplet, 2H), 7.79–7.92 (multiplet, 2H), 8.22 (doublet, 1H) and 8.24 (singlet, 1H), in close agreement with published data (Hanefeld et al. 1996; Bishop et al., 1999). High resolution mass spectrometry gave a mass of 331.181712 for the molecular ion (calculated mass for C_20_H_21_N_5_, 331.179696). The solid analog was stored at 4°C and dissolved at 25 mM in DMSO just before use.

### Yeast Strains:

*S. cerevisiae* strains used in these experiments (see Table 1) were derived from the wild-type w303 and were grown in appropriate synthetic media. The cdc28-as1/PPase 1-cs strain was constructed by crossing cdc28-as1 and PPase 1-cs and selecting haploid progeny that were unable to grow either in the presence of analog or at 11°C. The cdc28-as1/Sic1-GFP strain was constructed by transforming cdc28-as1 cells with the plasmid pRS303 cyc1-sic1-GFP, HIS3 (kindly provided by Dr. Matthias Peter) which enabled cells to express a Sic1-GFP fusion protein. For transformation, 100 mL of exponentially growing cells (OD_600_ = 0.4–0.8) were harvested by centrifugation at room temperature and resuspended in 5 mL of LiAc solution (1 mM lithium acetate, 10 mM Tris-HCl/ 1 mM EDTA pH 7.5). The cells were pelleted again and resuspended in 1 mL of the LiAc solution. To 100 μL of these cells, 1–2 μg plasmid DNA was added along with 10 μg salmon sperm carrier DNA. After brief vortexing, 700 μL PEG mixture (40 g polyethylene glycol 4000 dissolved in LiAc solution to a final volume of 100 mL) was added and the cells incubated at 30°C for 30 min in a water bath shaker at 180 rpm. Next, 70 μL DMSO was added, and the cells were gently mixed and heat shocked at 42°C for 15 min with gentle mixing every 5 min. After pelleting at room temperature, the cells were resuspended in 200 μL of SOS (0.3 g yeast extract, 0.6 g bactopeptone, 0.65 mL 1 M CaCl_2_ and 50 mL sorbitol in a total volume of 100 mL) and incubated 20 min at room temperature. 150 μL of these cells were plated on agar medium lacking histidine to select for cells carrying the plasmid. As a control, untransformed cells were plated on identical selective agar plates.

### Metaphase-Arrest; Treatments with Analog and α-Factor:

For metaphase arrest, cells growing exponentially at 28°C were treated for 3 h in one of two ways. In the first method, nocodazole was added to a final concentration of 12 μg/mL from a stock solution of 5 mg/mL in DMSO. This concentration was found in flow cytometry experiments to give the highest percentage of G2/M cells. In the second method, medium containing 30 μg/mL benomyl was prepared by heating the medium to boiling in a microwave oven, adding benomyl from a 30 mg/mL stock solution in DMSO and then cooling this medium to 28°C in a water bath. Cells were pelleted, resuspended in the benomyl-containing medium, and further treated with 15 μg/mL nocodazole. Additional nocodazole (7.5 μg/mL) was added at hourly intervals. The two methods gave identical results in experiments involving analog treatment, but the benomyl method gave a slightly higher percentage of G2/M cells.

For analog treatments, the stock solution of 1NM-PP1 was diluted in DMSO so that after addition to the cells the concentration of DMSO in the culture was 0.5% (v/v). For the cold-sensitive mutant cdc28-as1/PPase 1-cs, metaphase-arrested cultures were incubated at the non-permissive temperature (11°C) for 15 min to ensure equilibration at that temperature before adding 1NM-PP1. α-factor was added to a final concentration of 100 μM.

To view and quantitate rebudding or shmooing, 100 μL culture samples were fixed by adding 20 μL of a 3:1 mixture of methanol and acetic acid. Cdc28-as1/Sic1-GFP cells were viewed live by fluorescence microscopy.

### Protein Extraction and Analysis:

**Figure 1:**
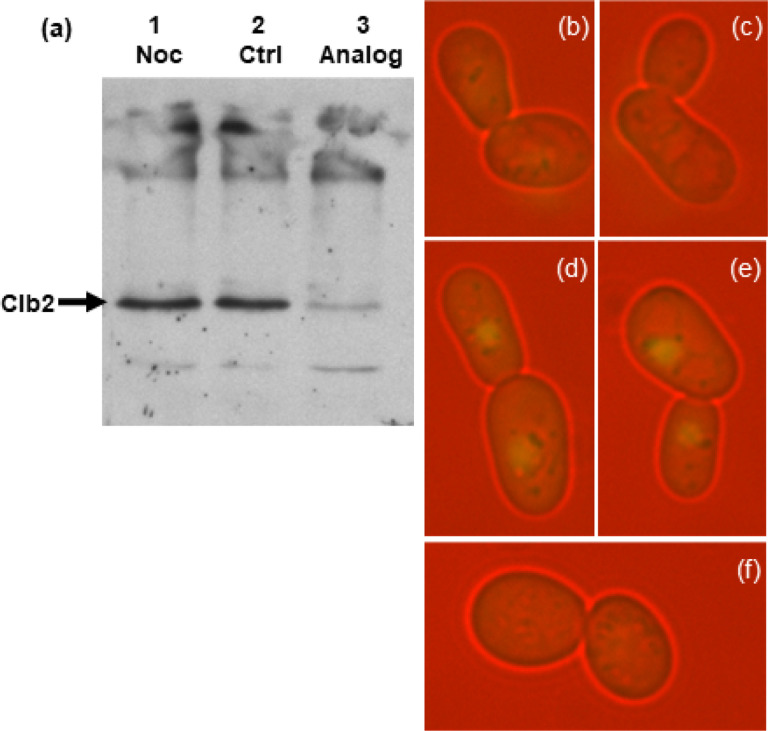
Degradation of Clb2 (Cyclin B) and stabilization of Sic1 following treatment of metaphase-arrested cdc28-as1 cells with 1NM-PP1. (a) Western immunoblotting with anti-Clb2 antibody: Lane 1 (Noc), cells arrested 3 h with nocodazole; lane 2 (Ctrl), cells arrested 3 h with nocodazole, then incubated an additional 1 h without 1NM-PP1; lane 3 (Analog), cells arrested 3 h with nocodazole, then incubated an additional 1 h with 1NM-PP1. (b)-(f) Fluorescence microscopy of cdc28-as1/Sic1-GFP cells: (b), (c) cells arrested with nocodazole for 3 h; (d), (e) cells arrested with nocodazole for 3 h, then treated with 1NM-PP1 for an additional 80 min; (f) a cell arrested with nocodazole for 3 h, then mock-treated for an additional 80 min. Bright nuclear fluorescence in (d) and (e) shows that Sic1-GFP has been stabilized, a sign that Cdc28 (Cdk1) has been inhibited. The bar indicates 5 μm.

For analysis of Clb2 (Fig. 1(a)), 3 mL of yeast culture was centrifuged and the pelleted cells were incubated on ice for 5 min. The pellet was then resuspended in 150 μL of 1.85 M NaOH, 1 M 2-mercaptoethanol and incubated 10 min on ice. Then 150 μL of 50% (w/v) trichloroacetic acid (TCA) was added and the sample incubated a further 10 min on ice. The sample was again centrifuged (15,000 × g, 2 min, 4°C) and the pellet resuspended in 1 mL acetone. The mix was centrifuged again (15,000 × g, 2 min, 4°C) and the pellet was resuspended with 5 μL 1 M Tris-HCl pH 7.5 and 140 μL of SDS gel final sample buffer. Protein concentrations were determined via Bradford assay, and 20 μg of protein was loaded onto a gel for each sample.

SDS polyacrylamide gel electrophoresis was run as described by Laemmli and Favre (1973) except using minigels containing 12% acrylamide and 0.32% piperazine diacrylamide (PDA, Pierce Chemical Co.) Proteins were electrophoretically transferred to PVDF membranes which were then blocked with 5% powdered milk in PBS. The primary antibody was rabbit anti-Clb2 (Santa Cruz Biotechnology) at 1:2000 dilution. The secondary antibody was HRP-conjugated goat-antirabbit IgG (Upstate). Detection was by Enhanced Chemiluminescence (ECL, Amersham Biosciences) using X-ray film.

## Results

### A. An Approach to Studying Cdk1 Inactivation and Mitotic Exit in *S. cerevisiae*

In previous investigations using metaphase-arrested mammalian cells, we have shown that inactivation or inhibition of Cdk1/Cyclin B (MPF) leads to exit from mitosis without chromosome segregation or cytokinesis (Paulson et al. 1996; Paulson 2007). Those results provided support for the hypothesis that Cdk1 inactivation is sufficient to trigger the transition from mitosis to G1-phase.

To carry out analogous studies on yeast, three things were required: First, a means to arrest cells in metaphase of mitosis; second, a way to inactivate MPF (Cdc28/Clb2); and third, methods to observe exit from mitosis.

**Figure 2:**
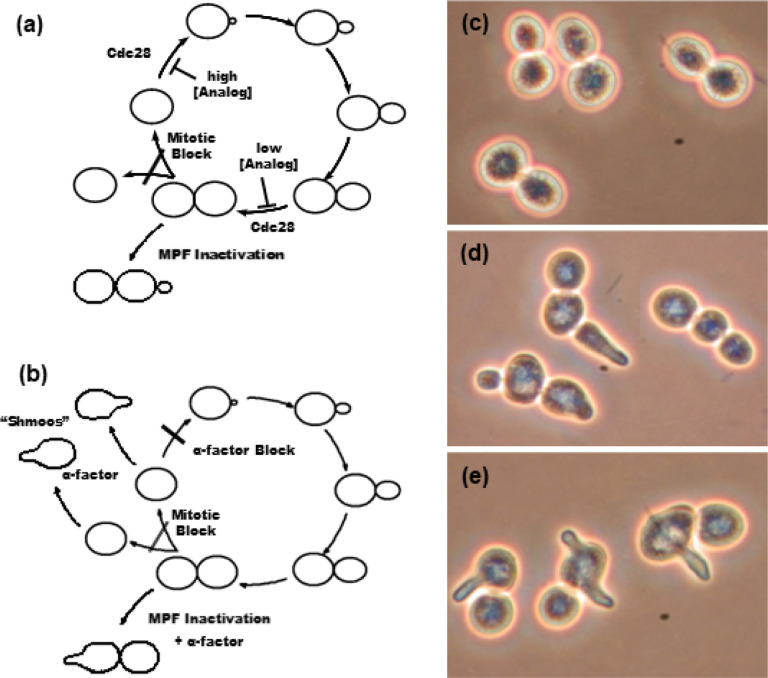
Morphological changes induced by analog treatment of metaphase-arrested *S. cerevisiae* cells carrying the *cdc28*-*as1* mutation. (a), (b) Schematics of the cell cycle explaining the predicted effects of the analog (1NM-PP1) alone (a) or in combination with α-factor (b). Note in (a) that Cdc28 (Cdk1) is required for rebudding, but a higher analog concentration is needed to block rebudding than to inactivate Cdc28 at mitosis (Bishop et al., 2000). (c)-(e) Phase contrast microscopy of (c) Cells arrested with nocodazole for 6 h; (d) Cells arrested with nocodazole for 3 h followed by treatment with 1NM-PP1 for 3 h. Note that the cells have not undergone cytokinesis but have rebudded; (e) Cells arrested with nocodazole for 3 h, then treated with 1NM-PP1 and α-factor for 3 h. Note the “shmoos” induced by the mating pheromone (α-factor). Shmooing can only occur in cells that are in G1-phase of the cell cycle. The bar indicates 5 μm.

In early experiments, *S. cerevisiae* were arrested in metaphase by treating exponentially growing cultures with 12 μg/mL nocodazole for 3 h at 28°C. This procedure typically gave 80–90% G2/M cells, as judged by microscopy (large “dumbbell” shaped cells; e.g., Fig. 2(c)). In later experiments, a combination of 15 μg/mL nocodazole and 30 μg/mL benomyl was used, with further additions of 7.5 μg/mL nocodazole at hourly intervals. This procedure typically gave 90–95% G2/M cells.

To inactivate MPF (Cdc28/Clb2), cells carrying the *cdc28*-*as1* mutation (Bishop et al., 2000) were treated with the bulky ATP analog 1NM-PP1 (see Materials and Methods). In the *cdc28*-*as1* mutant, the active site of Cdc28 has been engineered to make it specifically sensitive to inhibition by this compound (Bishop et al. 2000).

Observing exit from mitosis is more difficult in yeast than in mammalian cells. In mammalian cells, one can monitor reassembly of nuclear envelopes and decondensation of chromosomes by light microscopy (e.g., Paulson et al. 1996; Paulson 2007), but with yeast these criteria are not available because *S. cerevisiae* has a closed mitosis (the nuclear envelope remains intact throughout mitosis), and chromosome decondensation is difficult to observe because the cells and chromosomes are so small. Cytokinesis cannot be used as a criterion because inactivation of Cdk1 in metaphase-arrested cells triggers the transition to G1 without cell division (Paulson 2007).

We explored four potential criteria to judge whether exit from mitosis had occurred in *S. cerevisiae*: (1) rebudding; (2) induction of shmoos in MATa cells treated with α-factor; (3) degradation of Clb2; and (4) stabilization of Sic1. As will be discussed in the next section, rebudding and shmooing provide clear evidence that 1NM-PP1 treatment causes M-arrested *S. cerevisiae* cells to progress to G1-phase. Degradation of Clb2 and stabilization of Sic1 are consistent with mitotic exit and show that Cdc28 (Cdk1) has been inactivated, but do not directly show that cells have left mitosis. By themselves they leave open the possibility that some other event (in addition to Cdc28 inactivation) may be necessary to induce return to interphase.

Clb2 is the regulatory partner of Cdc28 that is degraded by the proteasome during exit from mitosis, thereby inactivating the kinase. Figure 1(a) shows that Clb2 is also degraded following treatment of metaphase-arrested cdc28-as1 cells with 1NM-PP1. Using western immunoblotting with an anti-Clb2 antibody, a strong Clb2 band is observed in extracts of nocodazole-arrested cdc28-as1 cultures (Fig. 1(a), lanes 1–2), but not in extracts of arrested cells that were treated for 1 h with 1NM-PP1 (Fig. 1(a), lane 3).

Sic1 is a protein inhibitor of Cdc28 (Mendenhall 1993; Mendenhall et al. 1995). During mitosis, Sic1 is phosphorylated by Cdc28/Clb2 which signals its proteolytic degradation (Sheaff and Roberts 1996; Verma et al. 1997; Feldman et al. 1997; Nishizawa et al. 1998). Sic1 is therefore not normally present during mitosis while Cdc28 is active, but after the kinase is inactivated it accumulates in the nucleus and remains there as the cell progresses to G1-phase.

To study Sic1 stabilization following treatment of M-arrested cdc28-as1 cells with 1NM-PP1, a plasmid encoding a Sic1-GFP fusion protein was inserted into the cdc28-as1 strain. When these cells were arrested in metaphase with nocodazole, no fluorescence was seen (Fig. 1(b), (c)), but after treatment for 1 hour with 1NM-PP1, Sic1-GFP fluorescence was observed in the nuclei of most cells (Fig. 1 (d), (e)). Nuclear GFP fluorescence was not seen in mock treated cells (Fig. 1(f)).

In summary, Clb2 degradation and Sic1 stabilization confirm that the Cdk1 kinase is inactivated when metaphase-arrested cdc28-as1 cells are treated with 1NM-PP1.

### B. Specific Inhibition of Cdc28 Induces Exit from Mitosis

Rebudding is a clear sign that a metaphase-arrested cell has exited mitosis because budding can only occur in a cell that has progressed through G1-phase. Similarly, shmoos can only be induced in G1-phase cells. A “shmoo” is a yeast cell that has undergone a morphological change, the appearance of a mating projection, that is for example induced in MATa cells by the mating pheromone α-factor (Merlini et al. 2013).

Figure 2 (a) and (b) illustrate schematically what is predicted to happen if a metaphase-arrested cdc28-as1 cell is treated with 1NM-PP1. In Fig. 2 (a), the cell leaves mitosis and enters G1-phase, but without dividing. It then progresses through G1 and initiates a new bud (lower left in Fig. 2(a)). Figure 2 (b) shows what we expect if the cell is MATa and both 1NM-PP1 and α-factor are present. Again, the cell exits mitosis to G1-phase without cytokinesis and shmooing is induced (lower left in Fig. 2(b)).

To test these predictions, cdc28-as1 cells were arrested in metaphase with nocodazole and/or benomyl and then treated with 1.5 μM 1NM-PP1. At various times, samples were fixed and viewed by phase-contrast light microscopy. After about one hour of incubation at 28°C, a high percentage of the analog-treated cdc28-as1 cells began to display tiny new buds which then grew over time. After 3 h treatment, the new buds were quite large (e.g., Fig. 2(d)). By contrast, metaphase-arrested cdc28-as1 cells incubated for 3 h without 1NM-PP1 showed little sign of rebudding (e.g., Fig. 2(c)), and wild-type (w303) cells also did not rebud, whether or not they were treated with 1NM-PP1 (data not shown).

The new buds (e.g., Fig. 2(d)) are nearly always distal to the old bud site, which is characteristic of diploid cells (Freifelder 1960). Although cdc28-as1 is a haploid strain, these cells have been induced to leave mitosis without dividing and thus have the same DNA content as a diploid cell. This may explain the bud site selection.

An aliquot of the same culture was treated with both 1NM-PP1 (1.5 μM) and α-factor (100 μM). After 3 h of treatment these cells underwent shmooing, again showing that they must have been induced to enter G1-phase (Fig. 2(e)).

### C. Effects of Different Concentrations of 1NM-PP1

*S. cerevisiae* has only one cyclin-dependent kinase, Cdc28 (Cdk1) (Nasmyth 1993), which is required both for the initiation and maintenance of the mitotic state and for initiation of budding and the onset of S-phase. The result in Fig. 2(d) is therefore somewhat surprising. One might expect that adding analog to inhibit Cdc28 would also block rebudding.

An explanation for this conundrum is suggested by the work of Bishop et al. (2000), who noted that a higher concentration of 1NM-PP1 is required to prevent entry into S-phase than to block entry into mitosis. That is, Cln-associated Cdc28 is less sensitive to 1NM-PP1 than Clb-associated Cdc28. Thus, there might be a “window” of analog concentration that is enough to induce exit from mitosis (or block entry into mitosis) but not enough to prevent rebudding.

To test this idea, aliquots of a metaphase-arrested culture growing at 28°C were treated with a range of different analog concentrations. After 3 h the cells were viewed in the phase contrast light microscope and the percentage that had rebudded (as in Fig. 2(d)) was determined for each analog concentration. At the same time, other aliquots of the culture were treated with the same range of analog concentrations and simultaneously with 100 μM α-factor. After 3 h of further incubation, the percentage of shmoos (as in Fig. 2(e)) was determined for each sample.

**Figure 3:**
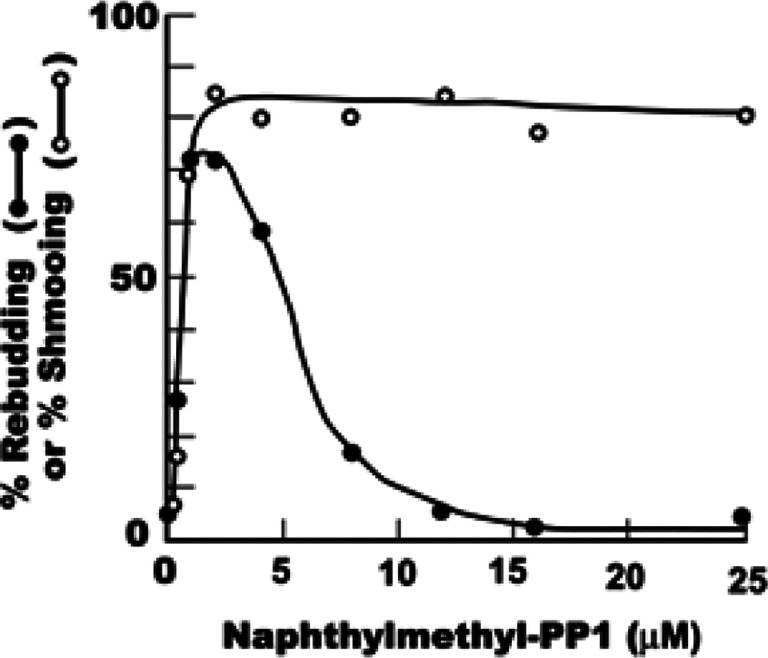
Percentage of rebudded cells and shmoos as a function of 1NM-PP1 concentration. Cells carrying *cdc28*-*as1* were arrested with nocodazole for 3 h and then treated for 3 h with various concentrations of 1NM-PP1, either with or without 100 μM α-factor. The graph shows the percentage of rebudded cells following treatment with 1NM-PP1 alone (●–●) and the percentage of shmoos following treatment with both 1NM-PP1 and α-factor (●–●). Note that high concentrations of 1NM-PP1 tend to repress rebudding (cf. Bishop et al, 2000) but do not affect shmooing.

The results are shown in Fig. 3. Clearly there is an optimal concentration of 1NM-PP1 (about 1.5–2 μM in this experiment) which produces the highest percentage of rebudded cells (Fig. 3, closed circles). Lower concentrations evidently do not inhibit Cdc28 sufficiently to induce exit from mitosis, whereas higher concentrations presumably induce exit from mitosis but then block rebudding. This is what one would predict if, as noted by Bishop et al. (2000), the function of Cdc28 in mitosis is more sensitive to the analog than its function in the G1- to S-phase transition.

This interpretation is confirmed by the second part of the experiment, employing α-factor. Optimal induction of shmoos (following treatment with both the analog and α-factor) also requires about 2 μM 1NM-PP1. But in contrast to the results with rebudding, the percentage of shmoos does not fall off at higher analog concentrations but instead levels off (Fig. 3, open circles), because shmooing, unlike rebudding, does not require Cdc28.

### D. Protein Phosphatase 1 is Required Downstream from Cdk1 Inactivation in Exit from Mitosis

Our ability to induce exit from mitosis by treating metaphase-arrested cdc28-as1 cells with 1NM-PP1 suggests a way to test whether particular enzymes or other proteins are required downstream from Cdk1 inactivation during mitotic exit. The idea is to combine the *cdc28*-*as1* mutation with a conditional mutation in the protein of interest, and then test whether metaphase-arrested cells of the double mutant exit mitosis when treated with 1NM-PP1 under non-permissive conditions.

Since Protein Phosphatase 1 (PPase 1) is known to dephosphorylate proteins during exit from mitosis in mammalian cells (see Discussion), we used this method to examine the role of PPase 1 in yeast. In *S. cerevisiae*, PPase 1 is encoded by a single gene GLC7, and we used the cold-sensitive mutation *glc7*-*129* (Bloecher and Tatchell 1999, 2000).

Wild-type (w303) cells, cdc28-as1 cells, and cells of the double mutant cdc28-as1/PPase 1-cs (*cdc28*-*as1/glc7*-*129*) were arrested in metaphase with nocodazole at 28°C and aliquots were treated with various concentrations of 1NM-PP1 at either 28°C or 11°C (the non-permissive temperature for *glc7*-*129*). After 3 hours at 28°C or 18 hours at 11°C, the percentages of rebudded cells in the various samples were determined in the phase contrast microscope.

**Figure 4:**
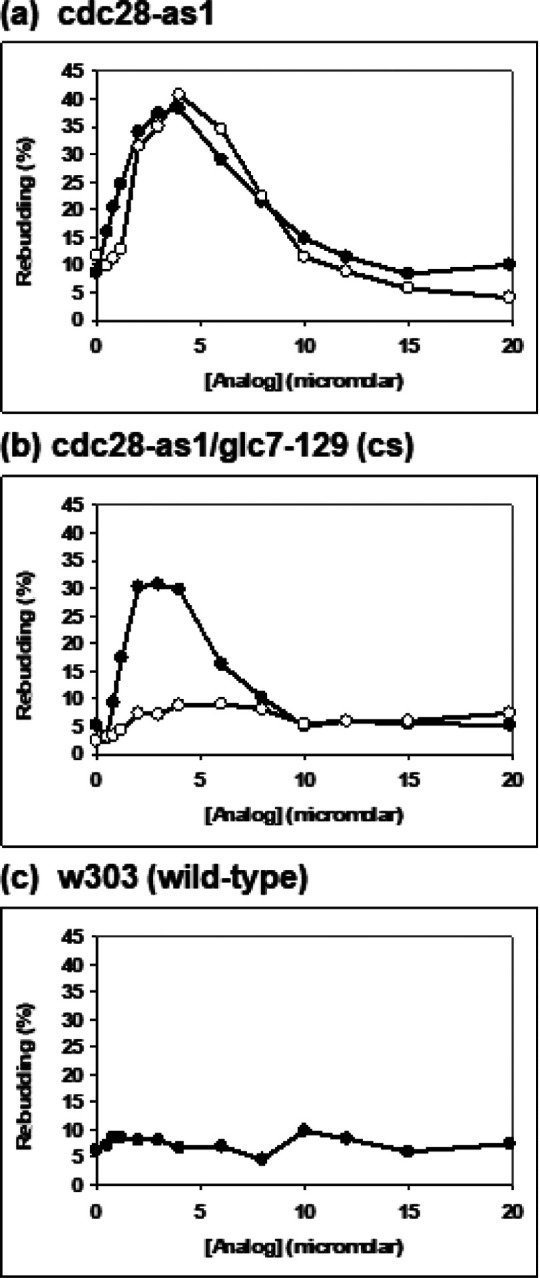
Rebudding does not occur when Protein Phosphatase 1 (PPase 1) is inactive. Cells carrying *cdc28*-*as1* and/or the cold-sensitive mutation *glc7*-*129* in PPase 1 (Glc7) (non-permissive temperature, 11°C) were arrested in mitosis and then treated with 1NM-PP1 either at 28°C or 11°C. (a) cdc28-as1; (b) cdc28-as1/PPase 1-cs (*cdc28*-*as1*/*glc7*-*129*); (c) w303 (parental wild-type strain). Incubation at 28°C (●–●) or 11°C (●–●).

Figure 4 shows the results of this experiment. With cdc28-as1, the results resembled those in Fig. 3 and did not differ between 11°C and 28°C (Fig. 4(a)). At 28°C, the double mutant cdc28-as1/PPase 1-cs gave similar results (Fig. 4(b), filled circles). However, at 11°C, cdc28-as1/PPase 1-cs did not show any significant rebudding (Fig. 4(b), open circles). As expected, there was no significant rebudding in wild-type (w303) cells (Fig. 4(c)).

A similar experiment was carried out using 1NM-PP1 plus α-factor and observing shmooing instead of rebudding. Since shmooing (unlike rebudding) is not affected by high concentrations of 1NM-PP1 (cf. Figure 3), 10 μM 1NM-PP1 was used for all treatments. Aliquots of metaphase-arrested cultures of w303, cdc28-as1, PPase 1-cs (*glc7*-*129*), and the double mutant cdc28-as1/PPase 1-cs (*cdc28*-*as1/glc7*-*129*), were treated with α-factor, either with or without 1NM-PP1. After 3 hours at 28°C or 18 hours at 11°C, the percentage of shmoos in each culture aliquot was determined.

The results in Table 2 show that significant amounts of shmooing are observed only when PPase 1 (Glc7) is active, the *cdc28*-*as1* mutation is present, and the cells are treated with both 1NM-PP1 and α-factor. With *cdc28*-*as1* alone, somewhat less shmooing occurs at the lower temperature, with 46.7% shmoos at 11°C versus 78.5% at 28°C. However, with the double mutant *cdc28*-*as1/glc7*-*129*, the amount of shmooing is greatly reduced at the non-permissive temperature, with 56.9% shmoos at 28°C but only 9.1% at 11°C.

## Discussion

### Inactivation of Cdk1 is Sufficient to Trigger Exit from Mitosis

As mentioned in the Introduction, it is well established that inactivation of MPF (Cdk1/Cyclin B) is *necessary* for cells to leave mitosis, but evidence indicates that Cdk1 inactivation is also *sufficient* to trigger mitotic exit, provided that all the factors required downstream from Cdk1 inactivation are functional. This hypothesis predicts that inactivation or inhibition of Cdk1 in metaphase-arrested cells will cause them to leave mitosis and enter G1-phase.

This prediction has previously been tested in several ways (reviewed by Paulson 2007). For example, treatment of metaphase-arrested mammalian cells with inhibitors of Cdk1 and other protein kinases (e.g., staurosporine; Gadbois et al. 1992) leads to nuclear reassembly and chromosome decondensation (Nakamura and Antoku 1993; Th’ng et al. 1994; Paulson et al. 1996; Hall et al. 1996; Potapova et al. 2006). Treatment of metaphase-arrested cells with sodium vanadate or other Cdc25 inhibitors does the same thing (Dunphy and Kumagai 1991; J.R. Paulson, unpublished work), presumably by allowing inhibitory phosphorylation of Cdk1 at Tyr-15 (Ajiro et al. 1996). Cdk1 inhibitors and vanadate also induce exit from meiotic metaphase II arrest in oocytes (Lee et al. 1999; Phillips et al. 2002). Induction of mitotic exit by heat-treatment of cells carrying temperature-sensitive mutations in Cdk1 has been observed in budding yeast (Ghiara et al. 1991), fission yeast (He et al. 1997), *Drosophila* embryos (Sigrist et al. 1995; Onischenko et al. 2005) and mouse FT210 cells (Paulson 2007).

Individually these results are not conclusive because the treatments used could have had secondary effects that were also required for leaving mitosis. For example, inhibitors of Cdk1 such as staurosporine may have inhibited other protein kinases and heat treatment of strains with temperature-sensitive Cdk1 may have affected other proteins whose inactivation was also required to leave mitosis. Nevertheless, collectively these observations constitute strong evidence for the hypothesis of sufficiency. It is very unlikely that all the methods used to inactivate Cdk1 would fortuitously have had the same secondary effects.

The initial aim of our work was to provide an even stronger test of the hypothesis of sufficiency using *S. cerevisiae* carrying the mutation *cdc28*-*as1*. In this mutant, Cdc28 has been engineered to be very specifically inhibited by the ATP analog 1NM-PP1 (Bishop et al. 2000).

Our results show clearly that when metaphase-arrested cdc28-as1 cells are treated with 1NM-PP1, the cells exit mitosis and enter G1-phase. This is demonstrated by rebudding (Figs. 2(d), 3 and 4) and by formation of shmoos when MATa cells are simultaneously treated with both 1NM-PP1 and α-factor (Figs. 2(e) and 3; Table 2).

Based on these results, we can say with great confidence that inactivation of Cdk1/cyclin B (MPF) is sufficient to trigger the transition from mitosis to G1-phase.

### A System to Study Factors Involved Downstream from Cdk1 Inactivation in Mitotic Exit

Our goal is to elucidate the events that occur and the enzymes and other proteins that are involved in the transition from mitosis to G1-phase. Inhibiting Cdc28-as1 with 1NM-PP1 provides a system for testing whether particular genes or proteins are required downstream from Cdk1 (Cdc28) inactivation in mitotic exit.

In this system, cells carrying the *cdc28*-*as1* allele are arrested in metaphase and treated with 1NM-PP1. At the same time, another protein suspected of a role in mitotic exit is inactivated in the cells, for example using a conditional mutation or a specific inhibitor of the protein. If these cells are unable to exit mitosis following 1NM-PP1 treatment, it will indicate that the protein of interest is essential downstream from MPF inactivation.

An advantage of this system is that a protein’s involvement downstream from Cdk1 inactivation can be observed independently of other roles it might play in mitosis, for example in spindle assembly, the anaphase signal, APC activation, cyclin degradation, and chromosome segregation. (Wu et al. (2009) noted a similar advantage in their study of protein dephosphorylation after treating metaphase Xenopus egg extracts with the Cdk1 inhibitor roscovitine.) The system can in principle be used to test any genes or proteins suspected of being required downstream from Cdk1 inactivation, provided that conditional mutations, conditional knockouts, or specific inhibitors are available.

### Protein Phosphatase 1 is Required Downstream from Cdk1 (Cdc28) Inactivation

We applied the system described above to Protein Phosphatase 1 (PPase 1) because it is known to be involved in mitosis in *S. cerevisiae* (e.g., Black et al. 1995; Zhang et al. 1995; Peggie et al. 2002; Bӧhm and Buchberger 2013) and because in higher cells it clearly plays a role in the M- to G1-phase transition. In higher eukaryotes it is involved in dephosphorylation of nuclear lamin proteins and nuclear reassembly (Peter et al. 1991; Thompson et al. 1997; Steen et al. 2000; Vagnarelli and Earnshaw 2012; Huguet et al. 2019, 2022), reassembly of nuclear pore complexes (Laurell et al. 2011), chromosome decondensation (Landsverk and Bollen 2005), and disassembly of kinetochores (Emanuele et al. 2008). It dephosphorylates histone H1 (Paulson et al. 1996), histone H3 (Hsu et al. 2000; Vagnarelli et al. 2011), retinoblastoma protein (pRb) (Ludlow et al. 1993; Nelson and Ludlow 1997; Nelson et al. 1997; Puntoni and Villa-Moruzzi 1997; Yan and Mumby 1999; Rubin et al. 2001), and other proteins (Wu et al. 2009; Mochida and Hunt 2012; de Castro et al. 2016; Holder et al. 2019, 2020).

Our results show clearly that in *S. cerevisiae*, PPase 1 (Glc7) is required downstream from Cdk1 (Cdc28) inactivation during exit from mitosis. In a strain containing both the *cdc28*-*as1* mutation and cold-sensitive Glc7 (*glc7*-*129*), rebudding is seen after treatment with 1NM-PP1 at the permissive temperature (28°C) but not at the non-permissive temperature (11°C) (Fig. 4(b)). Similarly, treatment of the double mutant with both 1NM-PP1 and α-factor leads to shmooing at 28°C but not at 11°C (Table 2). In a strain with *cdc28*-*as1* and wild-type Glc7, budding and shmooing occur normally at both temperatures (Fig. 4(a) and Table 2).

### Protein Phosphatases and Their Substrates in Mitotic Exit

As noted in the Introduction, many proteins are phosphorylated by Cdk1/cyclin B and secondary protein kinases at the onset of mitosis and dephosphorylated when cells leave mitosis. This leads to the question, which protein phosphatases are involved during mitotic exit?

Our results indicate that at least some essential protein dephosphorylations are catalyzed by PPase 1. We suggest that PPase 1 may in fact be the major protein phosphatase that acts downstream from Cdk1 inactivation in *S. cerevisiae*. Of course, other protein phosphatases are known to be involved in budding yeast mitosis including PPase 2A (e.g., Queralt et al. 2006; Alberghina et al. 2009; Grallert et al. 2015; Holder et al. 2019) and Cdc14 Phosphatase (e.g., Shou et al. 1999; Visintin et al. 1998, 1999).

It is claimed that the Cdc14 Phosphatase “likely dephosphorylates many, if not all, Clb-Cdk [i.e., Cdk1/Cyclin B] substrates” (Stegmeier and Amon 2004). However, its primary role may be upstream from Cdk1 inactivation. Although it dephosphorylates some factors required for DNA replication (Bloom and Cross 2007) and cytokinesis (Palani et al. 2012; Kao et al. 2014), most of the Cdk1 substrates Cdc14 is known to act on are part of the mechanism by which Cdk1/Cyclin B is inactivated. They include Cdc15, Cdh1, Lte1, Sic1, the transcription factor Swi5, and others (Shou et al. 1999; Visintin et al. 1999; Jaspersen et al. 1999; Jaspersen and Morgan 2000; Bembenek and Yu 2003; Stegmeier and Amon 2004; Akiyoshi and Biggins 2010; Bloom et al. 2011; Godfrey et al. 2015).

The system we have described could be used to test whether Cdc14, PPase 2A and other protein phosphatases are required downstream from Cdk1 inactivation. Furthermore, the system could be combined with phosphoproteome analysis (e.g., Touati et al. 2018) to gain information about the substrates of the protein phosphatases that act downstream from Cdk1 inactivation. For example, phosphoproteins that are not dephosphorylated after treatment of cdc28-as1/PPase 1-cs with 1NM-PP1 at 11°C could be substrates of PPase 1, but those that are dephosphorylated in that situation would likely be substrates of other protein phosphatases.

## Figures and Tables

**Table 1:** Yeast Strains Used in this Study^a^

Strain Designation	Genotype	Source
w303^b^	ade2-1, his3-11,15, leu2-3,112, trp1-1, ura3-1, can1-100	M. Peter
cdc28-as1	cdc28-as1, ade2-1, his3-11,15, leu2-3,112, trp1-1, ura3-1, can1-100	M. Peter
cdc28-as1/Sic1-GFP	cdc28-as1, ade2-1, his3-11,15, leu2-3,112, trp1-1, ura3-1, can1-100 pRS303:cyc1:sic1-GFP:HIS3	This study
PPase 1-cs	glc7-129, his3-11,15, leu2-3,112, ura3-52	K. Tatchell
cdc28-as1/PPase 1-cs	cdc28-as1, glc7-129, ade2-1, his3-11,15, leu2-3,112, trp1-1, ura3, can1-100	This study

a All strains MAT a

b Contains a bud4 mutation that causes haploids to bud with a mixture of axial and bipolar budding patterns (Voth et al. 2005).

**Table 2:** Percentage of Shmoos after Treatment with Analog and α-Factor, 11°C versus 28°C

Strain	28°C		11°C	
	−Analog	+Analog	−Analog	+Analog
w303 (wild-type)	1.2 ± 0.4	5.7 ± 0.9	1.8 ± 0.5	2.6 ± 0.6
cdc28-as1	0.9 ± 0.4	78.5 ± 1.7	0.6 ± 0.3	46.7 ± 2.0
cdc28-as1/PPase1-cs	1.1 ± 0.4	56.7 ± 2.0	0.0	9.1 ± 1.0
PPase1-cs	1.2 ± 0.4	3.9 ± 0.7	1.9 ± 0.5	2.6 ± 0.7

Error limits indicate 95% confidence levels (2σ).

## Abbreviations

Cdc28: Cdk1 in *S. cerevisiae*
Cdc28-as1: Cdc28 sensitive to the ATP analog 1NM-PP1
Cdk1: Cyclin dependent kinase 1
MPF: Mitosis Promoting Factor (Cdk1/cyclin B)
1NM-PP1: ATP analog 4-amino-1-*tert*-butyl-3-(1′-naphthylmethyl) pyrazolo[3,4-d]pyrimidine
PPase 1: Protein Phosphatase 1
SDS: sodium dodecyl sulfate
